# Nicolau Syndrome: An Unforeseen Yet Evadable Consequence of Intramuscular Injection

**DOI:** 10.1055/s-0041-1728652

**Published:** 2021-05-25

**Authors:** Prakash K. Sasmal, Ankit Sahoo, Pradeep Kumar Singh, Vikram VS

**Affiliations:** 1 Department of Surgery, All India Institute of Medical Sciences, Bhubaneswar, Odisha, India

**Keywords:** intramuscular diclofenac, embolia cutis medicamentosa, dermatitis, necrotizing fascitis

## Abstract

An intramuscular (IM) injection is one of the common routes for administering drugs, commonly analgesics and vaccines. Nicolau syndrome refers to the rapid-onset painful, extensive cutaneous discoloration progressing to necrosis and ulceration, reported after IM injections. This case report highlights a rare but avoidable complication of such injections. An elderly man presented with extensive cutaneous necrosis and discoloration over the buttocks extending to the thigh, within few days after receiving a single shot of IM injection of diclofenac. Management involved wound care, biopsy, and cultures with supportive antibiotics to control superadded infection. After multiple sittings of extensive surgical debridement, the wound showed signs of healing and was ultimately amicable for skin grafting in a month.

Health care workers need sensitization toward such a complication that can occur out of a routine procedure like an IM injection. They should follow standard IM injections techniques and take precautions to avoid this mishap, which adds to the patient's morbidity.

## Background

Tissue necrosis following intramuscular (IM) injections was termed Nicolau syndrome after being first reported by Nicolau, in 1925, in syphilis patients who received bismuth injections. 1 Later on, several reports came up with similar manifestations following IM injections of nonsteroidal anti-inflammatory drugs (NSAIDs), corticosteroids, penicillin, or local anesthetic agent. Although rare, this can cause substantial morbidity to the patient if not managed efficiently. Beginning as pain and pallor over the site, it rapidly transforms into an erythematous patch, livedoid reticular pattern, patchy hemorrhagic lesions, and blisters, followed by necrosis and ulceration. Pathogenesis, although multifactorial, predominantly involves intense vasospasm with extensive perivascular inflammatory infiltrates and vascular thrombosis. This case report draws attention to how a seemingly routine small needle prick to administer diclofenac can, at times, herald catastrophic consequences, thus adding up to the morbidity of the patient.

## Case Presentation

A 60-year-old man presented to the emergency room with the sudden onset of black discoloration of extensive patches of skin over buttocks extending to the thigh after receiving one dose of injection diclofenac IM in his left buttock 5 days back. A local health care worker administered the drug to relieve the patient from a transient episode of intense headache. The patient experienced severe burning sensation over that area which gradually turned red. He had applied ice pack compression over the site with the hope of getting some relief. The lesion progressed in subsequent days to form blisters, which ruptured and finally turned black. Over 4 to 5 days, it spread to the opposite buttock, the upper thighs, lower back, and the groin, giving it an odd appearance. The pain had subsided over time with resulting extensive gangrenous patches of skin. He had no complaints of fever or other signs of systemic infection. The person never had a similar experience previously despite having received IM diclofenac for various causes. He had no known history of any drug reactions or allergies in the past. He was a known smoker and ganja addict, consumed alcohol, and was without any known medical comorbidities.

**Fig. 1:**
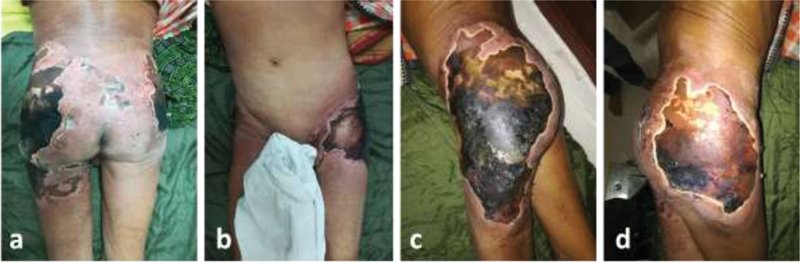
Extensive areas of necrosis covered with black eschar present over the bilateral gluteal region—( **a** ) bilateral buttock affected; ( **b** ) the lesion extending anteriorly on left side; ( **c** ) close view of left gluteal area showing necrosis, with desquamated areas and surrounding skin erythematous; and ( **d** ) close view of right gluteal area.

On examination, there were extensive areas of necrosis covered with black eschar present over the bilateral gluteal region and lateral upper thighs (left more than right) extending into the anterior aspect of left groin ( Fig. 1 ). The surrounding skin was erythematous and indurated with pus discharge at places from the margins. There was sparing of the genitals and perineum. All peripheral pulses (dorsalis pedis, popliteal, and femoral pulses) were palpable and bilaterally symmetrical, and no sensory or motor deficit noted over the lower limbs. No considerable delay in the capillary refill time was measured over the lower extremities. We did not have evidence of skin discoloration or ulceration elsewhere. Examination of abdomen and cardiovascular and respiratory systems revealed no abnormalities.

## Investigations

Blood parameters revealed raised white cell counts of 14,500 per microliter of blood with predominant neutrophilia and raised erythrocyte sedimentation rate (ESR) of 30 mm/h. All parameters, including blood sugar, liver function, renal function, coagulation profile, and viral serological markers, were normal. Cultures from the wound pus revealed growth of *Klebsiella* species and *Acinetobacter* species, and appropriate antibiotics were started.

## Treatment

**Fig. 2:**
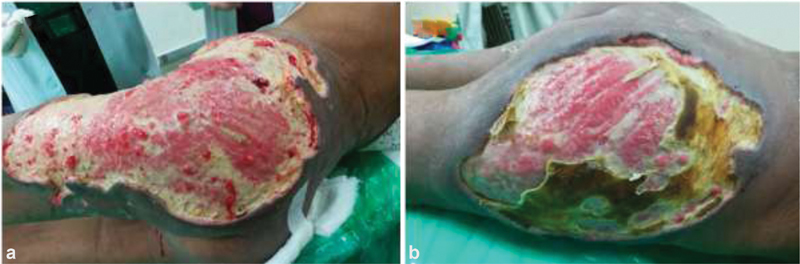
Post debridement in phases reveals necrosis of the subcutaneous tissues, sparing the underlying muscles—( **a** ) left gluteal region and ( **b** ) right gluteal region.

**Fig. 3:**
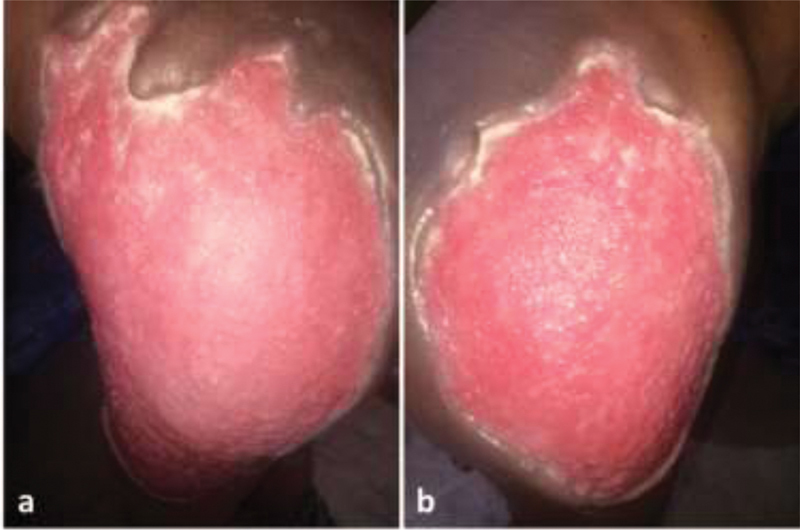
Healthy granulation tissues after 6 weeks—( **a** ) left gluteal region and ( **b** ) right gluteal region.

As extensive necrosis had already set in, the treatment focused mainly on local wound care and supportive medical management. The patient underwent extensive debridement of the necrotic tissues in consecutive sittings to reveal the subcutaneous tissues' necrosis sparing the underlying muscles ( Fig. 2 ). The granulation tissue filled up the defect after serial debridement and dressing of the affected site for nearly 6 weeks ( Fig. 3 ). We managed to cover the residual raw areas with healthy granulation tissues with split-thickness skin grafts with successful take-up.

## Outcome and Follow-up

Biopsy from the margin of the lesion showed dermal inflammatory infiltrates of lymphocytes and neutrophils. Vessels demonstrated infiltration of inflammatory cells into the walls and fibrin thrombi. The subcuticular layer also showed necrosis with inflammatory infiltrates.

The patient is doing well after one year of follow-up, with the grafted area being healthy.

## Discussion

Nicolau syndrome, also ascribed as embolia cutis medicamentosa or livedoid dermatitis, dates back to 1924 when Freudenthal and Nicolau, in 1925, first observed this cutaneous phenomenon in syphilis patients who received IM injections of bismuth salts for their treatment. 1 It refers to a rare adverse reaction following IM, and occasional subcutaneous and intra-articular, injections of certain drugs, characterized by the occurrence of an immediate acute inflammatory response in the skin and subcutaneous tissues followed by necrosis and eschar formation. 2 At times, necrosis can extend into the deeper muscle layers and get complicated by a superadded bacterial infection.

Various reports of similar reactions occurring after injection of numerous other substances have pointed out that this phenomenon could be unrelated to the drug administered. There are incidences of Nicolau syndrome reported after injecting drugs such as antibiotics (especially sulfonamide, procaine, and benzathine penicillin), vaccines (varicella, DTP), antihistaminics (diphenhydramine), NSAIDs (diclofenac sodium, ketoprofen, piroxicam), local anesthetics, corticosteroids (triamcinolone), vitamin B _12_ , sedatives (chlorpromazine and phenobarbital), interferons, coumarin, sclerosing agents, and many more. 3 4 Application of cold packs over the area, as commonly done, has been observed to aggravate the lesion's extent. 5

Several hypotheses are trying to explain the pathogenesis of this syndrome. May be several factors must be simultaneously contributing to the cascade of events. Intra-arterial, periarterial, or perineural administration of drugs initiates secondary vasospastic mechanisms by sympathetic nerve stimulation, inducing ischemia and eventually necrosis. 6 Intravascular viscous drug deposits further aggravate the condition by embolic occlusion of the vessels, as proved by traces of intra-arterial bismuth deposits found in biopsy specimens of patients described by Nicolau. 7 8 Superadded inflammatory infiltrates damage the endothelial lining and contribute to the thrombosis. 2 Administration of NSAIDs specifically worsens ischemia by inhibiting cyclooxygenase mediated prostaglandin production. 9 Physical obstruction of vessels by lipophilic agents that penetrates is also a proposed mechanism of injury. 10 In the present case, the necrotic patch was extensive involving the buttocks and thigh, which cannot be explained solely due to local vascular spasm or thrombosis. The previously hypothesized allergic or immunologic mechanism could not explain the absence of recurrence when these patients received the same injectable substance.

The lesions manifest early after the IM administration of the drug. Acute-onset local vasospasm leads to intense pain and pallor over the injected site, which progresses to become erythematous indurated macule with livedoid patches and branching pattern seen over the skin. Blisters appear next which rupture to leave behind an ulcerated surface which is subsequently covered by eschar. If small, the eschar eventually sloughs off with resulting ulcer which takes a prolonged course, ultimately terminating in an atrophic scar. 8

Often the serum ESR and c-reactive protein (CRP) levels are raised due to the presence of extensive inflammation. These patients usually have an unremarkable Doppler study as it mostly affects the smaller vessels at the cutaneous level. Biopsy of these lesions characteristically shows necrosis of the epidermis along with eccrine glands, extending into the underlying layers with extensive perivascular leucocytic infiltration, thrombosis of medium- and small-sized vessels of reticular dermis, and extravasation of erythrocytes, without the evidence of vasculitis. 4 8

There are few differentials to be kept in mind while evaluating these lesions. The condition often mimics necrotizing fasciitis, a life-threatening streptococcal or mixed infection with anaerobes, of the subcutaneous tissue following surgery or penetrating injury. 11 Suspicion of cutaneous embolization of atrial myxoma should arise in patients with existing cardiac comorbidities or those with acral injuries presenting with symptoms of dyspnoea and chest pain. Also, cutaneous cholesterol emboli can result in similar lesions following endovascular interventions in atherosclerotic patients. Vasculitis predisposing to such ulcers is also a possibility. 4 No history of cardiac ailments, endovascular procedures, cardiopulmonary symptoms, or evidence of vasculitis ruled out these possibilities in our patient. Hoigne syndrome is a pseudoanaphylactic reaction caused by vascular occlusion due to large crystals of penicillin salts seen after IM injections. 12 Treatment of these lesions is no different than other wounds. Local wound care with analgesia, infection prevention and control, application of topical steroids, and thrombolytic agents depending on the extent of lesions are mostly useful. Treatment depends on whether necrosis has set in or not. For those without necrosis, anticoagulant therapy and regular follow-up may be beneficial. 13 Extensive necrosis and ulceration, as in our case, mostly requires debridement and covering the defect with suitable grafts. Subcutaneous heparin, intravenous steroids, and pentoxifylline have yielded promising results. 8 14 Topical lactocalamine lotion with oral anti-inflammatory drugs have also proven effective in early lesions without necrosis. 15 Negative pressure wound therapy can also achieve better results and fasten wound healing after adequate debridement. 9

Nicolau syndrome is often complicated by myositis, abscesses, nerve palsy, myonecrosis, limb ischemia, and sepsis. 16 Compartment syndrome, though rare, has also been reported as a sequel in few cases, one of whom eventually required a below-knee amputation. 17 18 Fortunately, our patient did not have such complications.

Preventing the occurrence of Nicolau syndrome remains the cornerstone of management. Simple precautions and recommended technique of IM drug administration can eliminate the possibility of this dreadful adverse event. The upper outer quadrant of the gluteal region or midlateral portion of quadriceps femoris is ideal for IM injections due to lack of significant nerve bundles and vessels in this region. The abdominal wall is the preferred site for subcutaneous injections. 2 The Z-track method should be mandatorily applied while administering IM injections in adults. The needle track leaves a zig-zag path after withdrawing, thereby sealing the drug in the muscle layer, preventing extravasation into the overlying subcutaneous layers and minimizing chances of irritation and inflammation. The skin overlying the chosen site is pulled laterally or downwards, and the needle injected perpendicular inside. Upon confirmation of position, the drug is administered slowly and the needle removed at 90° after a brief pause. The taut skin is then released to create a zig-zag path. If aspirate yields blood, then the drug and needle must be discarded, and another site chosen. Massaging the area can lead to irritation and, hence, should be avoided. 19

Another essential point is that the needle length should be adequate to reach the muscle layer, especially in obese patients, and different sites should be chosen for administering multiple injections.

The occurrence of intense pain and burning sensation with skin discoloration should raise a suspicion of an impending Nicolau syndrome in the clinician's mind. One should refrain from applying ice packs to the injection site.

## Learning Points

- Nicolau syndrome is an avertible adversity; health care personnel, especially nurses and paramedics, must be made aware of this dreadful phenomenon.
- The occurrence of intense pain and burning sensation with skin discoloration should raise a suspicion of an impending Nicolau syndrome in the clinician's mind. One needs to refrain from applying ice packs.
- Timely diagnosis, proper wound care, and infection prevention are crucial to prevent dramatic repercussions of this complication.
